# Saving time, signaling trust: Using the PROMOTE self-report screening instrument to enhance prenatal care quality and therapeutic relationships

**DOI:** 10.1016/j.pecinn.2022.100030

**Published:** 2022-03-23

**Authors:** Heidi Preis, Clare Whitney, Christina Kocis, Marci Lobel

**Affiliations:** a Department of Psychology, Stony Brook University, Stony Brook, NY 11794, USA; b Department of Obstetrics and Gynecology, Renaissance School of Medicine, Stony Brook University, Stony Brook, NY 11794, USA; c School of Nursing, Renaissance School of Medicine, Stony Brook University, Stony Brook, NY 11794, USA

**Keywords:** Psychosocial screening, Prenatal care, Qualitative implementation research

## Abstract

**Objectives:**

Comprehensive screening of psychosocial vulnerabilities and substance use in prenatal care is critical to promote the health and well-being of pregnant patients. Effective implementation of new screening procedures and instruments should be accompanied by an in-depth investigation to assess their feasibility and impact on care delivery.

**Methods:**

In 2020, following implementation of the Profile for Maternal and Obstetric Treatment Effectiveness (PROMOTE) an innovative self-report screening instrument developed for outpatient prenatal clinics in the U.S., we conducted individual interviews and focus groups with twenty-two midwives, nurse practitioners, and obstetric residents focused on the PROMOTE and its impacts on care delivery. We used interpretive description for the qualitative analysis of the interviews.

**Results:**

Five themes were identified: Guiding Time Efficiently: “The Time I Don’t Have,” Preventing Missed Care, Signaling Trustworthiness, Establishing Trauma-Informed Foundations, and Promoting “Honest” Patient Disclosure.

**Conclusion:**

Interviews suggest that patient completion of the PROMOTE before the medical encounter helps reduce previously reported barriers, is more time-effective, and makes history-taking easier. It also facilitates the patient-provider relationship**.**

**Innovation:**

Findings offer insight into the breadth and depth of clinical impact resulting from the PROMOTE, and provide guidance for the implementation of such tools to optimize health outcomes.

## Highlights

• Comprehensive self-report screening in the first prenatal appointment is feasible
• Such screening using the PROMOTE can guide providers’ time more efficiently
• Aligned with trauma-informed care, it prepares patients to discuss sensitive topics
• It helps facilitate relationships among patient, provider, and practice community

## 1 Introduction

Prenatal care is a window of opportunity to detect vulnerabilities and offer treatment. Screening for psychosocial vulnerability and substance use is recommended for all patients in their first prenatal visit as this offers an opportunity for care providers to identify at-risk patients, counsel, and refer them to social and behavioral health specialists, a process known as Screening, Brief Intervention, and Referral to Treatment (SBIRT). To effectively implement screening of vulnerabilities that can affect the care and health outcomes of pregnant people, there is a need to understand the experiences of prenatal care providers in conducting comprehensive screening. The current research presents an in-depth analysis of the experiences of interprofessional prenatal care providers (i.e., nurse practitioners, midwives, obstetric residents) in implementing the Profile for Maternal and Obstetric Treatment Effectiveness (PROMOTE), a new self-report instrument to conduct comprehensive screening for psychosocial vulnerabilities and substance use [1].

### 1.1 Psychosocial screening in prenatal care

Substance use (e.g., tobacco, alcohol, opioids) and psychosocial vulnerabilities (e.g., high stress, lack of social support, residential instability) increase the risk of maternal physical and mental health morbidities and adverse perinatal outcomes including preterm birth and small size for gestational age [[2], [3], [4]]. Causes of these adverse outcomes are rooted in biomedical mechanisms such as teratogenic effects of substances or impacts of stress hormones on the body, but such outcomes are also affected by salutary maternal behaviors such as exercise and adequate prenatal care and deleterious behaviors such as polydrug abuse [5]. Modifying psychosocial circumstances that contribute to adverse perinatal health outcomes is key to promoting the health of mothers and their infants and reducing adverse outcomes. This is why leading organizations such as the Health Resources and Services Administration (i.e., Healthy Start programs funded by the Bureau of Maternal and Child Health) [6] and other obstetric and pediatric associations [2,7] recommend regular screening for mental health difficulties (e.g., depression, trauma), substance use, interpersonal violence, and suicide risk, and apply the SBIRT framework. SBIRT, which includes direct questioning by a health care provider, usually focuses on identification of substance use [8]. Yet comprehensive screening should include theoretically pertinent and clinically meaningful constructs such as stress, pregnancy planning, social support, financial condition, and psychiatric comorbidities that have been demonstrated to affect modifiable health outcomes [9].

Pregnant people, with or without substance use disorders, usually see their obstetrics provider more often than their primary care provider which makes prenatal care an opportune time to detect and address various concerns that may affect pregnant people’s general health and well-being as well as their perinatal health. However, comprehensive assessment is typically not part of routine care, in part because of the lack of an appropriate assessment instrument. There is great need for a validated instrument acceptable to providers and to patients that will facilitate SBIRT across multiple vulnerabilities affecting pregnant people.

### 1.2 Providers’ perspectives on screening for psychosocial vulnerabilities and substance use

In various health care settings, including prenatal care, implementation of SBIRT protocols is challenging as it requires the participation of staff who may not be prepared for additional tasks, involves working with patients who may be reluctant to disclose vulnerabilities, and requires administrative infrastructure that can accommodate the screening and referral process (e.g., systematic documentation in medical records, billing and payment, integrated areas of healthcare)[10]. Since care providers are key stakeholders in facilitation of SBIRT, it is critical to understand the factors that influence their ability to conduct a comprehensive psychosocial screening.

By and large, prenatal care providers view substance use, interpersonal violence, and depression screening as an important part of care provision and a way to detect vulnerabilities [[11], [12], [13]]. However, several institutional and provider-related barriers to screening have been identified as factors that can impede the success of such screening. Some of these include lack of validated instruments that are specific to the prenatal population and inadequate time to conduct screening given the many tasks that prenatal care providers are required to accomplish during the first prenatal visit [12]. Providers also report discomfort related to directly questioning pregnant people; this discomfort stems from concern about being perceived as judgmental or intrusive, fear of offending, and embarrassment [14,15]. Some providers report not fully understanding the purpose of the questioning and feeling uncomfortable when difficulties are disclosed [16]. However, there is evidence that pregnant people find it acceptable to be asked about substance use or mental health difficulties [15,[17], [18], [19]], and patient disclosure is an important opportunity to discuss prenatal health and mental health [20,21]. Patient-driven barriers to conducting psychosocial screening have also been documented, including partial or non-disclosure, and mistrust from patients who are afraid of stigma and of having child protective services involved [12]. Relatedly, providers have highlighted barriers related to screening when a patient’s partner is present, which can also impede open disclosure [12]. While there is growing knowledge on implementation of SBIRT frameworks in prenatal care, very little research was conducted in U.S. context [22,23], and there is a need to understand the experiences of implementing a comprehensive psychosocial screening instrument and procedure.

### 1.3 The PROMOTE

In clinical practice, having a simple instrument to screen for substance use risk and assess psychosocial vulnerabilities is extremely valuable. The PROMOTE is an instrument created for this purpose [1]. It is designed to comprehensively assess a wide array of vulnerabilities while alleviating screening barriers that often arise in prenatal care (e.g., provider difficulties in directly questioning patients, time constraints [24,25]). The PROMOTE is a page and a half long and includes nineteen single-item questions informed by clinical and theoretical evidence about the impacts of psychosocial vulnerability on prenatal care and health. Its development was spearheaded by behavioral scientists (removed for blind review) from the Department of Psychology with expertise in assessment in collaboration with obstetricians specializing in substance use and with nurse practitioners [1]. Each of the single-items assesses a different construct related to social determinants of health and psychosocial vulnerabilities such as financial insecurity, stress levels, social support (partner and family), healthy behaviors, and racial and ethnic identity, obstetric questions such as pregnancy planning, and timing of pregnancy discovery, and substance use questions such as the NIDA Quick screen [26]. Items are rated by patients on different response scales (e.g., Yes/ No, duration, frequency, magnitude) so that items are easily interpretable to providers without the need to calculate scores. For example, patients are asked “Since you became pregnant, did any major life events (break-up, moving, death of someone close, etc.) happen in your life?” with a No / Yes option followed by a prompt to indicate what that event was. As another example, patients are asked: “How much stress do you currently have in your life?” with a request to circle a response ranging from 1= very little to 5= very much. For those who endorse using substances, there are additional in-depth questions such as time of substance use start, being in substance use treatment, and use of injection drugs. The PROMOTE systematically collects relevant clinical information reported by a patient on a multitude of vulnerabilities, thus reducing the chance that vital aspects of a patient’s background will be overlooked.

Patients are asked to come to the clinic fifteen minutes before the set appointment time and among other documents given before the first prenatal visit (e.g., insurance and privacy waivers, the Edinburgh Postnatal Depression Scale), they are asked to complete the PROMOTE in paper form while in the waiting room. Once completed, the PROMOTE is viewed by a nurse practitioner, midwife, or obstetric resident that attends the first prenatal care visit for all patients who receive care at (removed for blind review). During this visit, the PROMOTE is intended to serve as a basis for further discussion to improve health-related, shared decision-making and referral to additional complementary psychiatric, psychological, social, or addiction services [[27], [28], [29]]. The PROMOTE is scanned into the electronic health record.

The PROMOTE was implemented into clinical use in June 2019. By the time interviews with providers were conducted in February 2020, 1,351 patients had completed the PROMOTE. Prior to implementation of the PROMOTE, patients would only complete the Edinburgh Postnatal Depression Scale, and SBIRT was conducted by a Medical Assistant that included the Audit-C for alcohol use, a question about safety at home, and about tobacco use. However, compliance with this SBIRT process was sub-optimal. Preliminary findings indicate that the PROMOTE is acceptable to patients and results in little missing data [1]. At the time of this writing, analysis of reliability and validity of the PROMOTE is ongoing.

### 1.4 Study aims

Since providers are vital for the effective implementation of any new screening methodologies, their perspectives on the instrument and its clinical application are critical to ensure good practice. The aim of this qualitative analysis was to query prenatal care providers (midwives, nurse practitioners, and residents) about their experiences incorporating the PROMOTE into care delivery and their experiences of caring for pregnant people with psychosocial vulnerabilities and substance use. We sought to learn about providers’ perceptions regarding how the PROMOTE might influence their approach to care in order to inform clinical practice and improve SBIRT and subsequent perinatal health outcomes for pregnant people and their infants. The research question, “What can be learned from prenatal care providers using the PROMOTE tool to care for pregnant persons with vulnerabilities?” guided data collection and analysis.

## 2 Methods

### 2.1 Methodological design

Thorne’s methodological approach of interpretive description was used for this qualitative analysis. Interpretive description draws on principles and techniques rooted in various sociological traditions [[30], [31], [32], [33]]. Positioned within the constructivist paradigm, interpretive description guides investigators to approach inquiry from a critically reflective understanding as an entry point into the field. Interpretive description prepares investigators to generate knowledge oriented in clinical wisdom, with utility for applied clinical fields [[33], [34], [35]].

### 2.2 Participant selection

Study participants were prenatal care providers working with pregnant people at (removed for blind review) outpatient clinics where the PROMOTE was implemented. (Removed for blind review) is a large academic medical center that serves a racially/ethnically and socioeconomically diverse rural/suburban population including low and high-risk pregnant people and a specialized program for pregnant people with substance use. Participants were purposefully sampled to include various professions-- midwives, nurse practitioners, obstetrics residents-- at different career stages. The total pool of these obstetric providers that were involved in implementation of the PROMOTE includes 48 professionals. They were invited by (removed for blind review) to participate in the study via communication through Division Chairs and administrators. Those interested in participating received information about the research and all provided informed consent before participating. Of those who were contacted by (removed for blind review), no one declined to participate. The study was approved by the Institutional Review Board of (removed for blind review).

### 2.3 Data collection

Data were gathered through semi-structured in-person individual interviews and focus group conducted by (removed for blind review). The interviews and focus groups ranged in length from 15 minutes to 30 minutes. In total, there were six individual interviews and three focus groups that took place over three weeks between 2/19/2020 and 3/6/2020 (Table 1), eight months after implementation of the PROMOTE (one interview that was deferred to June 2020 due to the COVID-19 pandemic). The nature of the interviews - individual or focus group - was informed by practical scheduling considerations with clinician participants. All interviews were conducted by (removed for blind review), a behavioral scientist with background in program evaluation, who developed the PROMOTE and who did not have any supervising role over any of the interviewees. The same interview guide was used for the individual and focus group interviews, which consisted of questions regarding clinical interactions with patients with psychosocial vulnerabilities, using the PROMOTE in clinical care, and the content of the PROMOTE. The PROMOTE itself was also distributed during the interviews for the interviewees reference. A trained research assistant aided the moderation of focus groups, and transcribed and de-identified all audio-recorded interviews, which were securely stored on a password protected computer.

**Table 1:** Study participants.

Interview type	Profession	Years in practice
Individual interviews	4 nurse practitioners and 1 midwife	0.5-24
Focus group 1	6 midwives	5-20
Focus group 2	7 residents	PGY1-PGY4
Focus group 3	4 residents	PGY1-PGY4

Note: PGY- Post Graduate Year

### 2.4 Data analysis

Data were analyzed using an inductive approach consistent with the theoretical and epistemological aspects of interpretive description. (Removed for blinded review), a qualitative researcher unfamiliar with the study questions or PROMOTE development, conducted the analysis, reducing potential bias during this stage. Analysis required repeated immersions in the data, gaining familiarity with individual cases – or participant narratives – and abstracting relevant themes from within them in order to generate findings that characterize the phenomena through a coherent conceptual description of thematic patterns [[33], [34], [35]]. First-pass coding involved a gestalt read of the data and analytic memo generation, noting initial patterns, differences, and contradictions within the dataset as a whole. Second-pass coding employed an iterative constant comparative approach to identify consistent patterns among participant narratives, and abstracted themes from these patterns. Subsequent coding passes involved continually subjecting existing themes to refinements and challenges, seeking saturation [[33], [34], [35]]. Through each phase of coding, the memos, patterns, and themes were discussed and debated by both (removed for blind review) in order to achieve consensus on each, enhancing the confirmability and validity of the analysis. Ultimately, the analyses generated findings that characterize phenomena through a coherent conceptual description of thematic patterns [34].

## 3 Results

A total of 22 prenatal care providers participated in individual or focus group interviews, including four nurse practitioners, seven midwives and eleven obstetrics residents (Table 1).  Analysis of participants’ accounts revealed that using the PROMOTE had implications for practice beyond its intended purpose for SBIRT. While much of the interview questions focused on acceptability and feasibility issues, inductive accounts reveal use implications of the PROMOTE that went beyond the initial interview focus. Five interrelated themes are identified and summarized, with corresponding participants narrative examples in Table 2.

**Table 2:** Definitions and patient narratives of themes.

Theme	Definition & Participant Narratives
Guiding Time Efficiently: “The Time I Don’t Have”	Providers eliminate redundant questioning, making the initial prenatal care appointment more efficient; Providers redirect time during visits toward building deeper relationships with entire clinical teams – moving beyond the initial prenatal care appointment. “It takes 30 seconds less to look this over before seeing a patient.” (Midwife) “With time constraints it might be something that you know, you might have gone through very quickly with a patient and now you have something that’s a little more comprehensive, you know that you can look at and discuss.” (Nurse Practitioner) “It can be helpful that way to kind of a quick overview, you know, where I can kind of curtail the conversation and spend more time on something else.” (Midwife) “[The PROMOTE is] a jumping off point too. Like, ‘Oh you know while I was reviewing this form and I was just looking through these answers… Let’s talk about that.’” (Nurse Practitioner)
Preventing Missed Care	Providers capitalize on opportunities for care that would otherwise be missed due to the time-constrained nature of clinical interactions. “If they need treatment options, if they need uhm counseling, you know whatever it may be… if somebody needs or is revealing an issue [through the PROMOTE] then we’re discussing, we’re on a path that we might not have been on.” (Nurse Practitioner) “With all our intake forms, this does the best job of getting a social history because we don’t ask these specific questions, like, ‘Do you rent or own your home?’ kind of thing… So it is nice having a little more of that information.” (Midwife) “It’s good [to] think about some of these factors before it becomes an acute issue. I feel like sometimes you, you know, things are lost and the patient just pops up with that, that housing is an issue… It’s good to work on this from the beginning.” (Resident)
Signaling Trustworthiness	Providers tailor care conversations and interventions around holistic perspectives of patients as a signal to patients that the provider can be trusted. “These touch upon sensitive issues, so you don’t just glance over something like, ‘Oh, you’re taking iron.’ It’s more like, ‘Tell me more about this specific conversation.’” (Resident) “People take a minute and a breath and really look at these things and go ‘Oh, ok she wrote that down, probably should say something about that,’ or making sure I get her in a path she needs to go down or whatever.” (Midwife)
Building Trauma-Informed Foundations	Patients establish expectations for care conversations regarding sensitive clinical and social factors by completing the PROMOTE prior to the start of the visit. “[The PROMOTE] makes them feel a little bit more comfortable uhm knowing that these are questions that are potentially going to be asked instead of me completely blindsiding … I think it kind of provides a little more comfort and confidence um, you know, through the visit.” (Nurse Practitioner) It’s like a gateway into… ‘I see that you said that you have been given Narcan. Can you tell me more about that?’” (Resident) “Offer[s] a way for someone to write down answers that maybe they might not be comfortable saying or maybe as a provider if we see something that, um is concerning to us and they have family in the room, it might like start the conversation of like, maybe ‘When I do the exam can everybody step out?’ and you have the conversation with them privately.” (Midwife)
Fostering “Honest” Patient Disclosure	Patients have an additional opportunity and alternative medium for disclosing sensitive information; Providers address privacy concerns related to the presence of family or other companions during provider visits. “When I first started, I was actually surprised how many [patients] are very forthcoming and do fill [the PROMOTE opioid screening] out and are pretty uh, pretty good about this.” (Nurse Practitioner) “It depends on the patient. Some people are more guarded, some people have issues that we don’t know that they have. Like there’s no…there’s only so much that you can ask. Like if they don’t want to tell you, they don’t want to tell you.” (Resident) “Rather than like when you’re going through your social history, almost expecting to hear ‘no’s’ for things in front of people.” (Midwife)

Providers spoke about how integrating the PROMOTE into practice improved their interactions by allowing them to gain time in immediate care encounters as a result of reducing redundancy of questioning and they also described gaining time in future care encounters by preventing missed care in the first visit. Time is also guided more efficiently, allowing providers to progress through screening activities more quickly and onto interventions, referrals, and treatment according to patient needs. Providers noted that using the PROMOTE had a salient impact on building interpersonal relationships with patients. Importantly, integrating the PROMOTE into practice allows providers to signal trustworthiness to patients by following up on patient responses and aligning sensitive conversations with the trajectory of the therapeutic relationship. Additionally, the trauma-informed foundation established by offering the PROMOTE prior to the patient’s visit with their provider fosters trust in the patient-provider relationship. Finally, providers described how using the PROMOTE can affect patient “honesty” (the term used by providers) in a manner that can facilitate the development of trusting interpersonal relationships between individual patients and providers.

### 3.1 Guiding time efficiently: “The Time I Don’t Have”

Providers described scenarios in which using the PROMOTE allowed them to make the initial prenatal care appointment more efficient. Specifically, providers felt more able to tailor their care conversations to the specific clinical and social needs of patients when using the PROMOTE. In such situations, clinicians did not need to spend time asking general or even redundant questions and instead guided care conversations using the PROMOTE to quickly triage care needs before the provider visit begins, as expressed in the reflection below:

I can look at it before, you know, before I go in, I think it, you know… cut[s] down on some of the time I don’t have to necessarily go through all of this, you know. And I’m like, okay, I have an idea of, I might still ask, you know, really quickly, you know, “Do you smoke?” or whatever. But I already kind of know, you know at least what they’ve written. (*Midwife)*

In effect, the PROMOTE serves to reduce the amount of time spent on information gathering and history-taking, which more quickly advances the focus of a visit to assessment, care planning, and intervention. Clinicians also conceptualize the PROMOTE as a “jumping off point,” as one nurse practitioner put it, from which they can assess patient needs and pursue deeper conversations about only those patient answers that warrant follow-up.

Providers highlighted the importance of the PROMOTE in building relationships between individual patients and the entire clinical team by guiding time efficiently. According to providers, creating more time for relationship-building rapport during care visits becomes increasingly important within the care setting of provider-rotated care. While rotating visits among various providers in a practice helps to increase the likelihood patients will prenatally meet with the provider who will be attending their birth, providers note that this approach to care also comes with disadvantages for establishing a connected relationship between a single provider and patient. Providers described time that would otherwise be spent on repetitive history-taking or lost to addressing the consequences of missed care. Time, that when using the PROMOTE, can be redirected toward building deeper relationships with entire clinical teams – moving beyond the initial prenatal care appointment during which the PROMOTE is completed.

As one provider detailed, the PROMOTE becomes increasingly important for the care of patients who do not yet have established relationships with the clinical team:

So maybe it will flag you know like having more one-on-one time, maybe it might be more comfortable, especially for patients who maybe are new to our practice and don’t know us or don’t have the history, are able to answer these questions this way instead of face-to-face. (*Midwife)*

By flagging issues from the start of care, the PROMOTE can support providers in directing time during a visit toward getting to know and establishing trust with patients, and it can support clinical teams in building stronger relationships with individual patients.

### 3.2 Preventing missed care

Providers described the role that the PROMOTE plays in capitalizing on opportunities for care that would otherwise be missed due to the time-constrained nature of clinical interactions. Providers also endorsed the importance of identifying and addressing clinical and social factors early on in care. Without the PROMOTE serving as a comprehensive screener, providers may have risked missing important social care needs and subsequent conversations to address such needs, as one provider illustrates:

It’s a reminder to make sure that those things are addressed and to address them and to have those conversations, I think… it gives you a chance to have a conversation… that may in the past have not been discussed. *(Nurse Practitioner)*

Using the PROMOTE allows providers the advantage of delivering quality care in a preventive – rather than tertiary – manner by identifying issues and avoiding missed care opportunities from the start of care.

### 3.3 Signaling trustworthiness

Providers pointed to the ways in which the PROMOTE facilitated holistic care by signaling to patients that the provider can be trusted. As one midwife described, using the PROMOTE allows clinicians to gain a holistic perspective of a patient – and their clinical and social needs – around which providers can tailor care conversations and interventions:

I feel like [the PROMOTE]… helps us uh establish a better relationship with the patient… because I think, I think, they feel like “Wow, you’re taking the time to ask me these questions or to look at this thing I filled out, uhm, and address things you think are concerning on there.” And I think that builds trust. *(Midwife)*

By demonstrating their attention to issues identified by patients themselves, providers can build trust while addressing important care needs. Ultimately, providers view the PROMOTE as enhancing not only the quality-of-care delivery, but also the depth and strength of provider-patient relationships.

### 3.4 Establishing trauma-informed foundations

Importantly, providers note the trauma-informed impact of integrating the PROMOTE into patient care. For example, by filling out the PROMOTE prior to the start of the visit, patients have the opportunity to establish expectations for care conversations regarding issues that they may not otherwise recognize as relevant to their obstetric and pregnancy needs. As one resident described, this can provide a trauma-informed “heads up” for patients to prepare them for potentially sensitive conversations with their providers:

[Patients] may not necessarily realize that these are all things we’re gonna be looking at too, and it kind of gives them a heads up that these are some things we’re gonna be asking. And they can almost like mentally prepare before meeting the provider by answering the questions. (*Resident)*

Indeed, providers value the way in which the PROMOTE serves as “a gateway” into sensitive issues. In situations where sensitive or potentially traumatizing topics will be discussed, a trauma-informed approach can enhance patient comfort by allowing them an opportunity to psychologically prepare for such discussions.

### 3.5 Promoting “honest” patient disclosure

Providers emphasized the importance of patient honesty and disclosure for quality care delivery. For example, providers noted how the PROMOTE can help to “get a really good and honest history from [patients],” as one resident put it, and mitigate factors hindering patient disclosure during care interactions. As a nurse practitioner described, the PROMOTE can inform the provider before the visit of any concerning medical history ahead of time, which allows them to create an environment in which the patient will feel more comfortable discussing aspects of their clinical or social history “truthful[ly]” with their providers:

Say I’m prepping a chart, um I’m going through [the PROMOTE] and getting her histories before meeting the patient you know. And I see that she’s had active heroin use in her last pregnancy you know. You know I’ll just be as direct as I can with that: “I know you have this history.” They tend to be very truthful in those moments, ‘cause they know that I know. (*Nurse Practitioner)*

Providers highlighted the tool’s ability to offer an additional opportunity and alternative medium for disclosing sensitive information as well as its utility for addressing privacy concerns related to the presence of family or other companions during provider visits.

## 4 Discussion and conclusion

### 4.1 Discussion

Interviews with prenatal care providers offer evidence regarding the utility of the PROMOTE to facilitate psychosocial screening in the first prenatal care visit. The interviews indicate that this innovative screening instrument is usable and can enhance the effectiveness and efficiency of the visit. Providers described the ways in which gained and guided time intersects with patient-provider honesty and trust to enhance relationships for care. Interpersonal relationships are built between patients and providers using the PROMOTE by guiding time efficiently, preventing missed care, signaling trustworthiness, establishing a trauma-informed foundation, and promoting patient “honesty”. Findings corroborate previously documented perceptions of prenatal care providers regarding the importance of psychosocial and substance use screening [[11], [12], [13]] and interviewees agreed that to deliver comprehensive care, there is a need to see the ‘whole person’ and know their psychosocial background to address issues that might affect their care, pregnancy, or well-being. Having patients complete the PROMOTE may reduce the risks of vulnerabilities not being detected and help providers when direct questioning is not a well-developed skill. In addition, providers need to receive training and supervision to master the screening process and learn how to use their intuition and clinical judgement during the SBIRT process [13,14,36].

Feasibility of the PROMOTE indicates that it can help overcome barriers to screening that have previously been identified, such as time constraints. Existing research suggests that a main impediment to implementing SBIRT models into prenatal care arises from the length of the first visit which includes taking an extensive medical history, pregnancy examination, patient education, and orientation to practice and prenatal care. In these busy first prenatal care visits, additional tasks of psychosocial and substance use screening could be burdensome [12]. The PROMOTE, which is completed by patients before their medical encounter, essentially saves time for the provider. If integrated properly into the workflow (i.e., before the medical encounter), it saves time from the first visit itself and for future visits. However, this requires organizational support and provider acceptance [37].

Having the PROMOTE completed by patients was discussed also as a means to improve open disclosure. Previous research indicates that patients favor self-report over direct questioning [38] and that self-report instruments yield greater disclosure compared to direct questioning or disclosure to the provider [39,40]. Providers using the PROMOTE mentioned that it offers an additional medium for patients to report substance use or other vulnerabilities and threats. Similarly, providers mentioned the benefits of the PROMOTE to facilitate disclosure, by signaling the need to speak with a patient privately, without a family member or significant other present, based on the responses a patient enters in the PROMOTE.

Additional aspects of trauma informed care were brought to light by participants in relation to the PROMOTE workflow. Similar to what has been reported previously, providers reported that the self-report screening helped ‘open the door’ to sensitive discussions, making it easier for both provider and patient [41]. Providers noted that it helps establish rapport between a provider and patients when the provider uses their intuition [13] and addresses the patient responses in the PROMOTE, making their patient feel seen, and demonstrating that that their difficulties matter. This corroborates findings from a study with vulnerable prenatal and postpartum patients who reported the importance of being listened to, feeling like they are being taken seriously, and being met with empathy [42].

### 4.2 Innovation

The PROMOTE is a novel screening instrument and procedure that expands SBIRT frameworks to conduct a comprehensive psychosocial assessment in prenatal care. The content of the PROMOTE, which includes an array of vulnerabilities, allows providers to see the ‘whole’ patient and identify difficulties that otherwise might not be asked or reported. Based on our findings, the PROMOTE also helps establish a trustworthy patient-provider relationship and trauma-informed care practice. The self-report administration also saves provider interviewing time and is especially valuable when providers are inexperienced or uncomfortable questioning pregnant people. In addition, a user-friendly, self-administered instrument can help overcome patient-driven barriers such as being embarrassed by providers’ reaction or reluctance to disclose when a partner can overhear responses.

While there is wide endorsement of the need to screen for substance use and psychosocial vulnerability in prenatal care among U.S. healthcare organizations [2,7,43], and efforts are being made to identify optimal assessment measures [44,45], it is clear that implementation is far from ideal [46]. The current investigation of the implementation of comprehensive screening instruments in the U.S. prenatal health care context is novel as very few such studies have been conducted in the U.S. [22,23]. Moreover, the qualitative method used to assess the implementation produced innovative findings regarding care delivery that extend beyond acceptability, barriers, and facilitators.

The limitations of this study suggest fruitful directions for future inquiry. First, study participants were limited to divisions within an obstetric department of a single institution. While the current findings can likely inform practice and policy at other obstetric departments of academic medical institutions, future research should investigate what can be learned from prenatal care providers using the PROMOTE tool to care for pregnant persons with vulnerabilities in other healthcare settings such as private clinics, or serving distinct or specialized populations of pregnant people. An additional consideration to examine is utilizing the PROMOTE in states with mandatory reporting policies for substance use in pregnancy. It will also be important for future inquiry to include the perspectives of professionals not included in this study’s sample. For example, capturing the perspectives of new-to-practice midwives, later career physicians, and other clinicians involved in the care of pregnant persons with vulnerabilities such as registered nurses, medical assistants, social workers, and other professionals can expand the applicability of findings to a wider audience. Finally, having the developer of the PROMOTE collect all of the qualitative data could have potentially introduced bias into focus groups and interviews.

### 4.3 Conclusion

This qualitative research indicates that the PROMOTE is a useful self-report tool to assist prenatal care providers conducting comprehensive screening of patients’ psychosocial vulnerabilities and substance use. Patient completion of the PROMOTE before the medical encounter helps reduce previously reported barriers, is more time-effective, and makes history taking easier. In addition, it helps facilitate relationships between the provider, patient, and even community. Study findings illustrate the breadth and depth of benefit to prenatal health care providers and their patients resulting from implementation of the PROMOTE, a comprehensive, self-report psychosocial screening tool that can easily be incorporated into routine care to improve care delivery and patients’ health and well-being.

## Declaration of Competing Interest

The authors report no conflict of interest. Research reported in this manuscript was supported by 10.13039/100000026National Institute on Drug Abuse under award number R21DA049827. The content is solely the responsibility of the authors and does not necessarily represent the official views of the National Institutes of Health.
